# Mesocellular Silica Foams (MCFs) with Tunable Pore Size as a Support for Lysozyme Immobilization: Adsorption Equilibrium and Kinetics, Biocomposite Properties

**DOI:** 10.3390/ijms21155479

**Published:** 2020-07-31

**Authors:** Agnieszka Chrzanowska, Anna Derylo-Marczewska, Malgorzata Wasilewska

**Affiliations:** Department of Physical Chemistry, Institute of Chemical Sciences, Faculty of Chemistry, Maria Curie-Sklodowska University in Lublin, M. Curie-Sklodowska Sq. 3, 20-031 Lublin, Poland; annad@hektor.umcs.lublin.pl (A.D.-M.); malgorzata.seczkowska@umcs.pl (M.W.)

**Keywords:** mesocellular silica foams, adsorption equilibrium, adsorption kinetics, protein/silica biocomposites, microscopic analysis, thermal analysis

## Abstract

The effect of the porous structure of mesocellular silica foams (MCFs) on the lysozyme (LYS) adsorption capacity, as well as the rate, was studied to design the effective sorbent for potential applications as the carriers of biomolecules. The structural (N_2_ adsorption/desorption isotherms), textural (SEM, TEM), acid-base (potentiometric titration), adsorption properties, and thermal characteristics of the obtained lysozyme/silica composites were studied. The protein adsorption equilibrium and kinetics showed significant dependence on silica pore size. For instance, LYS adsorption uptake on MCF-6.4 support (pore diameter 6.4 nm) was about 0.29 g/g. The equilibrium loading amount of LYS on MCF-14.5 material (pore size 14.5 nm) increased to 0.55 g/g. However, when the pore diameter was larger than 14.5 nm, the LYS adsorption value systematically decreased with increasing pore size (e.g., for MCF-30.1 was only 0.27 g/g). The electrostatic attractive interactions between the positively charged lysozyme (at pH = 7.4) and the negatively charged silica played a significant role in the immobilization process. The differences in protein adsorption and surface morphology for the biocomposites of various pore sizes were found. The thermal behavior of the studied bio/systems was conducted by TG/DSC/FTIR/MS coupled method. It was found that the thermal degradation of lysozyme/silica composites was a double-stage process in the temperature range 165–420–830 °C.

## 1. Introduction

The biomolecules adsorption (i.e., proteins, enzymes, pharmaceuticals) on liquid/solid interfaces is a common and complex phenomenon, important in its prominent role in biology, medicine, biotechnology, and food processing applications. Therefore, it is very important to fully understand mechanisms of adsorption processes of the compounds showing biological activity at different interface boundaries, as well as their practical and potential application as modified biological materials (BioSS—Biological Surface Science) [1,2].

The adsorption capacity and selectivity depend on several factors, including the protein properties (size, shape, charge, structure, stability, isoelectric point, and unfolding rate), the process conditions (solution pH, ionic strength, temperature), and especially the physicochemical properties of materials (surface porosity, morphology, hydrophobicity, heterogeneity, electric charge) [3,4,5,6,7]. For the large biological molecules immobilized on a porous support, the selection of optimal adsorbent is of great importance. In the case of mesoporous materials with pore sizes comparable with the biomolecule diameter, the internal loading of biomolecules should be lower due to the sieving effects. For materials with a pore diameter greater than the size of the adsorbed molecules, a possibility of penetration of biomolecules into mesopores and stronger interactions in the confined space results in higher adsorption. There is some evidence in the literature that the adsorbent pore diameter, which is at least two times larger than the adsorbate molecular diameter, enables full access to the internal space of mesoporous materials [8,9]. Thus, many efforts were undertaken to tune the mesopore size and structure of the well-ordered mesoporous molecular sieves (MMSes) and mesoporous silica nanoparticles (MSNs) to promote their bioapplications in catalysis, separation, disease diagnostics or as biosensors [10,11,12,13,14,15]. Many reports focused on usage of the most popular MMSes (MCM-41, SBA-15, MCM-48, MCM-21, MSE, FSM) as the efficient supports for the immobilization of various enzymes (cytochrome C, lysozyme, bovine serum albumin, ovalbumin, myoglobin, hemoglobin, trypsin, lipase, chloroperoxidase), to find the optimal size relation between mesopores and molecular dimensions of biomolecules for better control of adsorption process [16,17,18,19,20,21,22,23,24,25].

Among several types of high ordered mesoporous silica materials available for macromolecules immobilization, mesocellular silica foam (MCF) is one of the most suitable adsorbents for protein adsorption and separation, because of its unique structural properties (high surface area ~500–1000 m^2^/g, large pore volume ~2 cm^3^/g, large pore size up to 50 nm, uniformity of pore structure, high adsorption capacity, and especially the possibility of precise adjusting the structure and particle morphology). There are only a few reports regarding adsorption of myoglobin, lysozyme (LYS), bovine serum albumin (BSA), ovalbumin (OVA), chloroperoxidase, trypsin, a-amylase, glucoamylase on the MCF silica support [26]. For example, Russo et al. [27] studied the influence of surface functionalization on the adsorption capacity of BSA and LYS on the MCF silica with 31.5 nm pores. Essa et al. [28] found that adsorption of myoglobin on MMSes with differential porosities is relatively high for the MCF silica (pore size 14 nm) and SBA-15 (pore size 6.2 nm) in comparison to other silicas (MCM-41 pore size 3.1 nm, MSE pore size 6.0 nm, CNS pore size 14.7 nm). Han et al. [29] studied that the catalytic activity of chloroperoxidase enzyme on MCF (pore size 15 nm) and observed that it is higher than on MCM-48 (pore size 3.2 nm) and SBA-15 (pore size 4.2 nm and 7.0 nm). In our previous work, we found that MCF material with larger pores (pore size 10 nm and 16 nm) shows higher adsorption uptakes towards BSA and OVA proteins which can penetrate its internal structure to a larger extent [30]. However, there is still a lack of deeper analysis of the pore size – adsorption value relations in a wider range of pore diameters.

In the paper, the wide analysis of the dependence between the MCF pore size and specific surface area, and lysozyme adsorption capacity and rate is presented to optimize the adsorbent structural characteristics with regard to adsorbate uptake and density. It was found that there is an optimum relation between pore diameter and protein size in order to attain the maximum adsorption or surface density with regard to possible applications. Thus, the studies are important to obtain the material with desired porosity, which can be used as the effective support for biomolecules. We aim at the analysis of the influence of MCF pore size in a wider range of pore diameters on the adsorption capacity and rate of lysozyme, which is a comparatively small protein enzyme. A lysozyme from chicken egg white (LYS) was chosen as a model protein enzyme for immobilization on MCF supports due to its physicochemical properties and biological function: high structural stability (due to four disulfide bridges (S-S) and inflexibility between pH = 1.5 and 12; it makes lysozyme a fine model protein for interfacial adsorption in a wide range of pH); “hard model protein” which can undergo interfacial reorientations but does not unfold at an interface; small globular protein of molecular mass 14.3 kDa and hydrodynamic radius ~2 nm (LYS molecules adsorbed on the surface and inside MCF pores); positively charged at pH = 7.4 (electrostatic attraction between the protein and the silica material); strong antibacterial activity against Gram-positive organism, anti-inflammatory, immune-modulatory, and anti-tumor properties.

Several samples of MCF materials with tunable porosity and pore size were synthesized as carriers for immobilizing LYS molecules. The adsorption processes of lysozyme from phosphate-buffered saline (PBS) solutions in physiological conditions (pH = 7.4) on the series of MCF materials with different porosity were investigated. The equilibrium adsorption isotherms and kinetic dependences (concentration vs. time profiles) for the adsorption processes of biomolecules were determined. The dependences between pore diameter and kinetic rate were analysed. The acid-base character and surface charge density of the MCF supports covered by protein molecules were determined by using the potentiometric titration. Likewise, the structural, morphological, and textural properties of protein/silica composites were characterized employing the nitrogen adsorption/desorption isotherms, the Scanning Electron Microscopy (SEM), and Transmission Electron Microscopy (TEM). Additionally, the thermal stability and characteristics of the decomposition products of lysozyme/silica were studied by using a combination of the Thermogravimetric Analyzer (TG) with Mass Spectrometer (MS) or a Fourier Transform Infrared Spectrometer (FTIR). The applied powerful methods (TG/FTIR/MS) provided comprehensive information for understanding enzyme protein immobilization on the silica support.

## 2. Results and Discussion

### 2.1. Structure Characterization of MCF Supports

The porosity of the synthesized series of siliceous mesocellular foams (MCFs) was investigated by the nitrogen adsorption/desorption method. The comparison of nitrogen adsorption/desorption isotherms for three selected mesoporous MCF materials: MCF-6.4 with small pore size (*D_h_* = 6.4 nm, the average hydraulic pore diameter), MCF-14.5 with medium pore size (*D_h_* = 14.5 nm), MCF-30.1 with large pore size (*D_h_* = 30.1 nm) is presented in Figure S1A (Supplementary Material). The isotherms for the studied MCF adsorbents correspond to IV type according to IUPAC classification. They reveal well-defined H1 hysteresis loops with very steep and parallel adsorption and desorption branches at higher relative pressures [31,32]. The maximum adsorption is the smallest for MCF-6.4 material; however, it is comparable for MCF-14.5, and MCF-30.1 supports what is associated with similar large total pore and mesopore volumes of these materials. Moreover, the shift of hysteresis loops towards higher relative pressures for MCF-14.5 and MCF-30.1 carriers in comparison to MCF-6.4 is associated with larger pore diameters.

The parameters characterizing the textural properties of all synthesized MCF supports are compared in Table 1. It should be noted that the obtained MCF materials are characterized by a differentiated mesoporous structure concerning the values of specific surface areas, pore volumes, and pore diameters. All MCF materials show a well-developed porous structure with a high BET (Brunauer–Emmett–Teller) surface area varying from 250 to 720 m^2^/g, total pore volume 1–2 cm^3^/g, and a hydraulic pore diameter in the range 6–30 nm. As can be seen, the average pore diameters obtained from adsorption and desorption branches of isotherms using the Barrett–Joyner–Halenda (BJH) procedure are 6–38 nm and 5–30 nm, respectively. It is worthy to note that the pore sizes of synthesized MCF adsorbents are larger than the size of the lysozyme molecule (the hydrodynamic diameter ~4 nm). The specific surface area of MCF-6.4 (716 m^2^/g) is by ca. 3 times higher than for MCF-30.1 material. No micropores are detected for MCF-6.4, however, in the case of the other materials slight micropores are found which confirm the high quality of the obtained adsorbents with uniform porosity.

Pore size distributions (PSDs) for three selected materials calculated from the adsorption and desorption data using the BJH method are demonstrated in Figure S1B,C (Supplementary Material). The PSD plots for MCF-6.4 and MCF-14.5 show sharp peaks, suggesting that porous structure is fairly homogeneous with narrow pore sizes, however, for MCF-30.1 the pore size distribution is wider. The PSDs calculated from the adsorption and desorption data are shifted towards higher values of pore diameter as follows MCF-6.4 < MCF-14.5 < MCF-30.1.

### 2.2. Adsorption Equilibrium and Kinetics

**Figure 1:**
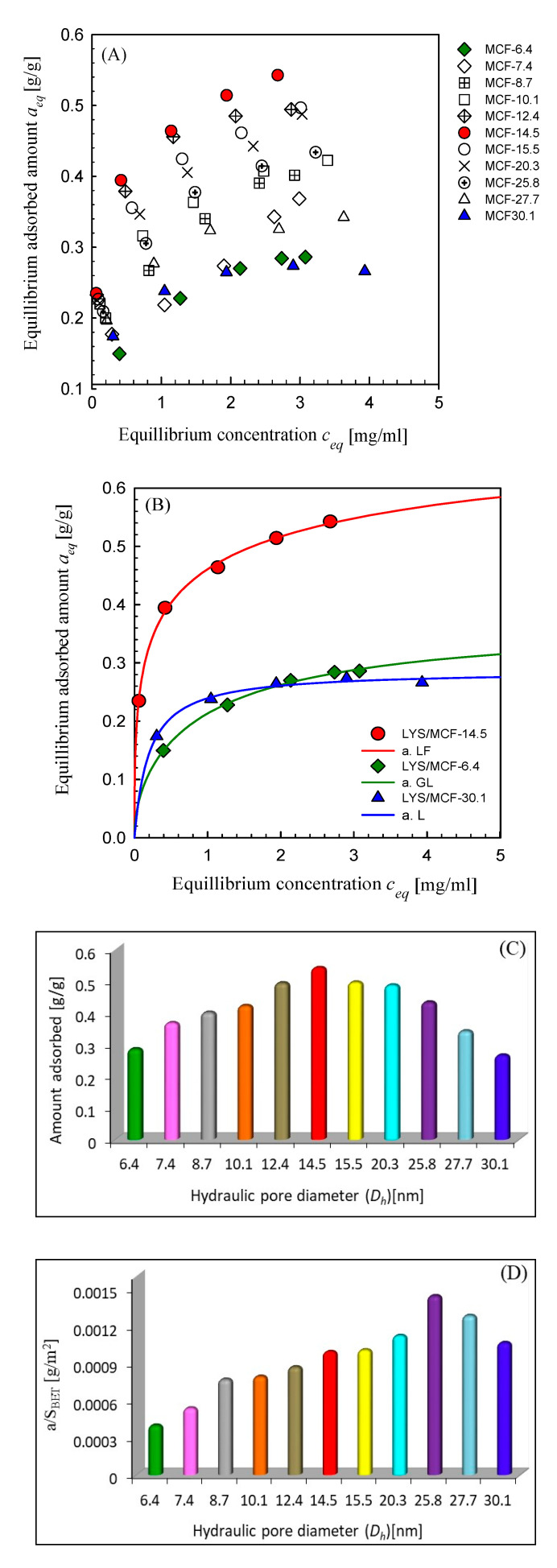
(**A**) Comparison of lysozyme (LYS) adsorption isotherms on mesoporous silica materials with different pore diameters; (**B**) LYS adsorption isotherms on three selected mesocellular silica foams (MCF) silica materials. Solid lines correspond to the fitted Generalized-Langmuir (GL) isotherm; (**C**) Relationships between the LYS adsorbed amounts, and (**D**) the LYS adsorption density versus the hydraulic pore diameter. Adsorption conditions: the initial concentration of protein solution (c_0_ = 5 mg/mL) in phosphate-buffered saline (pH = 7.4) at 25 °C.

In Figure 1A the adsorption isotherms of lysozyme are compared for all synthesized MCF supports. Moreover, in Figure 1B the experimental isotherms are compared for three selected materials with the medium, small, and large pore sizes. One can find that the strongest adsorption is observed for MCF-14.5. In the case of other materials of lower and higher pore sizes, a decrease of LYS adsorption is found. Moreover, the lowest adsorption is observed for MCF-6.4 and MCF-30.1, the supports with the narrowest and the most widened pores. This tendency is well presented in Figure 1C. Such a behavior may be correlated with the values of specific surface areas and pore sizes of investigated adsorbents. It is evident that the optimum pore size for lysozyme adsorption is around 14.5 nm. The other MCF materials with comparable specific surface areas, however, with different pore sizes (lower and higher than 14.5 nm) reveal weaker adsorption affinity. It may be explained by the relation of protein size and pore diameter, which in the case of MCF-14.5 and LYS is responsible for the increase of the adsorption forces. However, when we regard the adsorption surface density (adsorption/specific surface area) it occurs that the density maximum is shifted to MCF-25.8 (see Figure 1D). Thus, in the case of this material, the protein layer of the highest density is formed. We can compare our adsorption results with those obtained by other authors for the silica materials with pore sizes in the range of 3.9–19.2 nm. The lysozyme adsorption isotherms on SBA-15 with different pore sizes (9.8–19.2 nm) at pH = 10.6 have been reported by Santos et al. [33] the adsorbed amounts were equal to 0.2 g/g (pore size 9.8 nm), 0.28 (11.7 nm), 0.47 (17.3 nm) and 0.79 (19.2 nm). In another study, MCM-41 (pore size 3.9 nm) and SBA-15 (8.8 nm) were used and LYS adsorption capacity was estimated to be 0.19 and 0.38, and 0.41 and 0.48 g/g at pH = 6 and 10.5, respectively [34]. According to Moerz et al. [35], the amount of LYS adsorbed on SBA-15 (pore size 6.6 nm) at pH = 6 and 10.6 was: ~0.12 g/g (~8.5 μmol/g) and ~0.29 (~20.5 μmol/g), respectively; however, at pH~ 7.3, it was about 1.5 times lower (~13.5 μmol/g; ~0.19 g/g) than in the case of our material. The adsorption capacities characterizing our MCF materials are as follows (pH = 7.4): 0.29 g/g (pore size 6.4 nm), 0.42 (10.1 nm), 0.49 (12.4 nm), 0.55 (14.5 nm), 0.49 (20.3 nm). Considering the dependence of protein adsorption on solution pH and ionic strength we can state that our materials reveal good sorption properties.

For the analysis of these data, the Generalized Langmuir equation was used. As one can find the GL isotherm well fitted the experimental data. The parameters of this equation (and its special forms) are presented in Table 2. One can find that the optimized values of adsorption capacities (*a_m_*) agree quite well with the experimental uptakes, the observed small differences (lower theoretical *a_m_* values in comparison to experimental ones) result from the fact that the experimental systems did not yet reach equilibrium. For two systems LYS/MCF-14.5 and LYS/MCF-6.4, the values of heterogeneity parameters *m* and *n* lower than 1 indicate their high nonhomogeneity. However, for LYS/MCM-30.1 energetic homogeneity is observed (*m*, *n* = 1), which may be correlated with the largest pores of adsorbent and resulting in a more homogeneous distribution of protein molecules on the surface.

**Figure 2:**
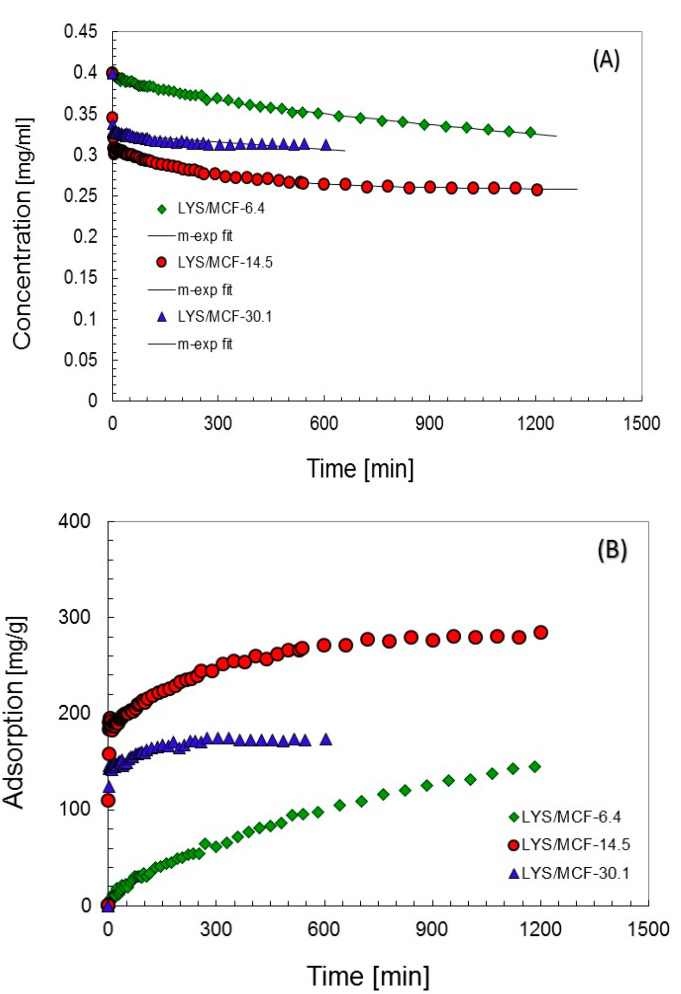
The comparison of concentration (**A**) and adsorption (**B**) profiles measured for lysozyme adsorption on MCF-14.5, MCF-6.4, and MCF-30.1 supports. Adsorption conditions: c_0_ = 0.4 mg/mL, T = 25 °C; pH = 7.4.

In Figure 2, the experimental concentration and adsorption profiles are compared for the selected MCF supports. The concentration vs. time curves was analyzed by using the multi-exponential (m-exp) equation (theoretical lines in Figure 2A) (the parameters of this equation are presented in Table 3), and the other kinetic equations and models (first-order (FOE), second-order (SOE), mixed-order (MOE), fractal first-order (f-FOE), fractal second-order (f-SOE), fractal mixed-order (f-MOE) equation, intraparticle diffusion model (IDM), McKay pore diffusion model (PDM)). The choice of m-exp equation for the description of experimental data was based on the lowest values of standard deviation SD. The optimization results for m-exp equation to other equations and models are compared in Table S1 (Supplementary Material) in which the values of relative standard deviations SD(c/c_0_) are given. Basing on the average SD values one can find that m-exp equation gives the best optimization results, for the PDM model the poorest results were found. However, it should be stated that in the case of the system LYS/MCM-6.4 the lowest SD values were found for two fractal models f-FOE and f-SOE (SD = 0.246% and 0.247%, respectively), but for m-exp equation SD is only slightly higher (SD = 0.337%).

In Table 3, the values of optimized m-exp eq. parameters characterizing adsorption kinetics are compared: the rate constant logarithms (log*k_i_*) and the *f_i_* coefficients determining a fraction of a solute adsorbed with rate *k_i_*, moreover, the adsorption half-time is presented. The quality of optimization results is confirmed by low standard deviation, SD, values (0.337–0.723%), and indetermination coefficient, 1-R^2^ (0.0027–0.021). The kinetic experimental systems are well optimized by 2 or 3 terms of m-exp equation. The values of log*k_i_* and *f_i_* parameters characterizing the stages of the adsorption process indicate that the initial stage is quicker for all systems investigated. It may be attributed to adsorption on the external surfaces which are accompanied by the second slower stage connected with adsorption in the pore system. Analyzing the experimental kinetic curves one can find that the adsorption process is the slowest for MCF-6.4 (*t_1/2_* = 358.7) with the narrowest pores which indicates some problems with the diffusion of protein molecules into the internal pore space. The range near adsorption equilibrium was attained in the shortest period for MCF-30.1 (*t_1/2_* = 0.5) which may be explained by easier diffusion of protein molecules into large pores. The adsorption process is slightly slower for MCF-14.5 (*t_1/2_* = 1.7) which means that the pore sizes of this material do not disturb protein diffusion. Moreover, the kinetic profiles for MCF-30.1 and MCF-6.4 achieve the comparable close to equilibrium adsorption values, however, for MCF-14.5 the equilibrium adsorption is higher, and these results are in accordance with batch experiment (Figure 2B).

### 2.3. Physicochemical Properties of Protein/MCF Composites

#### 2.3.1. Structural Analysis

The N_2_ adsorption/desorption measurements were used to determine semi-qualitatively whether the adsorbed biomolecules were situated inside the pores of the MCF materials and/or at the external surface. The nitrogen adsorption/desorption isotherms for pure supports (MCF-6.4, MCF-14.5, MCF-30.1) and biocomposites are compared in Figure S2A (Supplementary Material). The parameters characterizing the changes in the pore structure of MCF materials after protein adsorption are summarized in Table 4. It can be found in Figure S2A (Supplementary Material) that the amount of nitrogen adsorbed on biocomposite is decreased with the increasing amount of LYS (the LYS adsorbed amounts from solution are as follows: for MCF-6.4 (pore size 6.4 nm) is ca. 0.29 g/g, for MCF-14.5 (pore size 14.5 nm) increased to 0.55 g/g, for MCF-30.1 (pore size 30.1 nm) is ca. 0.27 g/g.

Furthermore, the analysis of structural properties (Table 4) of the MCF silica supports before and after LYS adsorption shows that the porosity parameters (*S_BET_,* the BET specific surface area; *V_t_,* the total pore volume; *V_mes_,* the mesopore volume; *D_hy_,* the average hydraulic pore diameter) estimated from N_2_ adsorption/desorption data are strongly reduced. The strongest reduction of pore characteristics is observed for LYS/MCF-14.5 biocomposite which is very well correlated with the highest adsorption of protein. It can be seen that for MCF-14.5 material with immobilized LYS the BET surface area decreased from 547 m^2^/g to 38 m^2^/g (93%). On the other hand, for LYS/MCF-6.4 and LYS/MCF-30.1 *S_BET_* values diminished by approximately 52% and 54%, respectively. Additionally, LYS adsorption leads to decrease of the total pore volume of approximately: 94% for MCF-14.5, 57% for MCF-6.4, and 73% for LYS/MCF-30.1. The values of average pore diameter (*D_av_*) and the mean hydraulic pore diameter (*D_hy_*) also decrease after protein adsorption. The reduction in the specific surface area, pore volume, and size after LYS adsorption observed for all MCF materials indicates that the protein molecules are adsorbed inside the mesopores of MCF material as well as on the external surfaces. The evolution of pore-size distribution functions obtained from BJH model for isotherm desorption branches for MCF-14.5, MCF-6.4, and MCF-30.1 after protein adsorption are presented in Figure S2B (Supplementary Material). One can find a strong decrease of the peak heights for all biocomposites in comparison to pure supports: 96% for MCF-14.5, 26% for MCF-6.4, 61% for MCF-30.1.

#### 2.3.2. Acid-Base Properties

**Figure 3:**
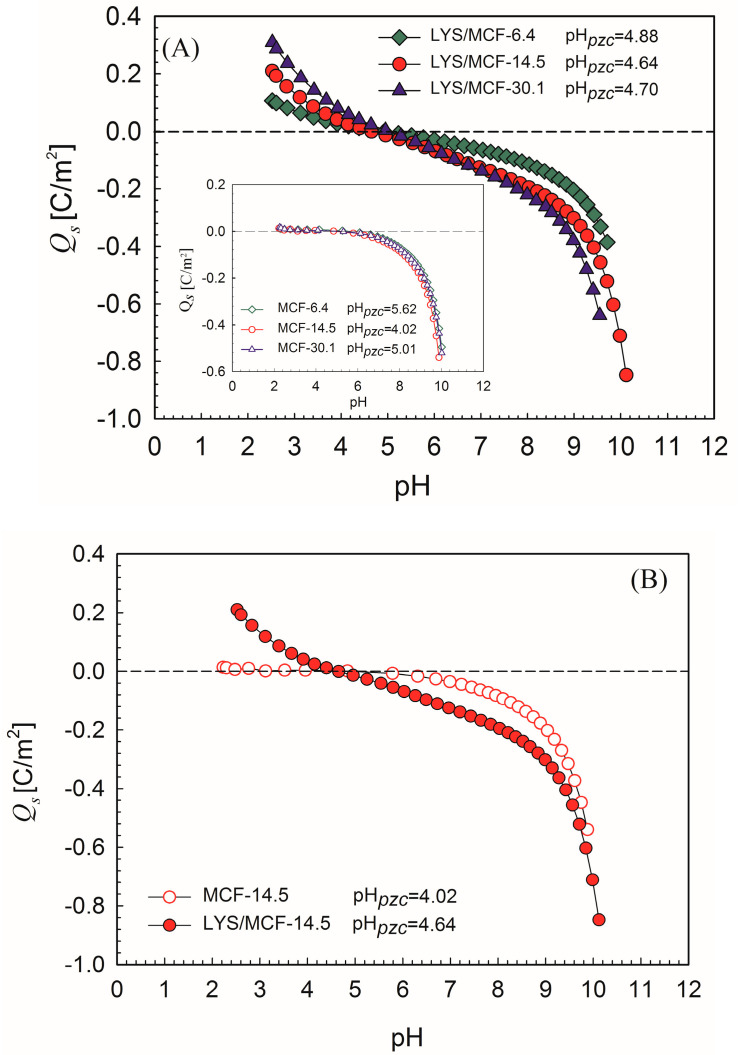
(**A**) Dependence of surface charge density, *Q_s_*, on pH for MCF-6.4, MCF-14.5, MCF-30.1 materials with immobilized lysozyme. Inset: dependence of *Q*_s_ vs. pH for pure MCF-6.4, MCF-14.5, MCF-30.1 silicas; (**B**) Surface charge density, *Q_s_*, for MCF-14.5 before and after LYS adsorption. The measurements were carried out by potentiometric titration for ionic strength I = 0.1 mol/L.

To evaluate the acid-base character, surface charge, and nature of the interactions between lysozyme and porous MCF the potentiometric titration measurements were applied. The surface charge density (*Q_s_*) curves as a function of pH calculated from the experimental data for the pure MCF materials and LYS/MCF systems are illustrated in Figure 3A,B.

As shown in Figure 3A (inset), for pure MCF supports with different pore sizes (MCF-6.4, MCF-14.5, and MCF-30.1), the point of zero charge, pH*_pzc_*, is in the range 4 to 5.6. As a result of protein adsorption, the change of biocomposites acid-base properties into amphiphilic is observed with pH*_pzc_* in the range 4.64–4.88 (Figure 3A). In the case of pure MCF-6.4, MCF-14.5 and MCF-30.1 their surfaces at experimental conditions (PBS, pH = 7.4) are slightly negatively charged with *Q_s_* ca. −0.05, −0.06, −0.04 C/m^2^, respectively (inset in Figure 3A). It is interesting to note that the surface charge density after LYS adsorption increases up to −0.08 C/m^2^ for LYS/MCF-6.4, −0.15 C/m^2^ for LYS/MCF-14.5 and −0.17 C/m^2^ for LYS/MCF-30.1 (Figure 3A,B). The presented results indicate the small negative surface charge for all studied MCF supports at pH = 7.4. Thus, regarding the positive charge of lysozyme at pH = 7.4, the electrical attraction between the silica surface and the protein molecule can be supposed. Considering high protein adsorption values, it should be assumed that the electrostatic attractive interactions play a significant role in the immobilization process [36]. In (Supplementary Material) the relation among the pore diameters and specific surface areas of MCF adsorbents and biocomposites, and the points of zero charge (pH*_pzc_*) is demonstrated in Figure S3. One can find that the acid-base character of the studied pure silica surface changes in comparison to the silica modified by LYS. Generally, as demonstrated in Figure S3 (Supplementary Material), for MCF silica materials after adsorption of lysozyme pH*_pzc_* stabilizes.

### 2.4. Thermal Analysis (TG/DSC/FTIR/MS)

The use of thermogravimetry (TG), derivative thermogravimetry (DTG), and differential scanning calorimetry (DSC) is a widely applied method for characterizing the thermal properties by measuring changes in physical and chemical properties (transition, dehydration, decomposition) during the heating process of materials. By enhancing temperature the protein, degradation is induced, leading to the removal of organic material from the inorganic support. The mass loss during thermal decomposition corresponds to the amount of protein adsorbed on the support [37,38].

**Figure 4:**
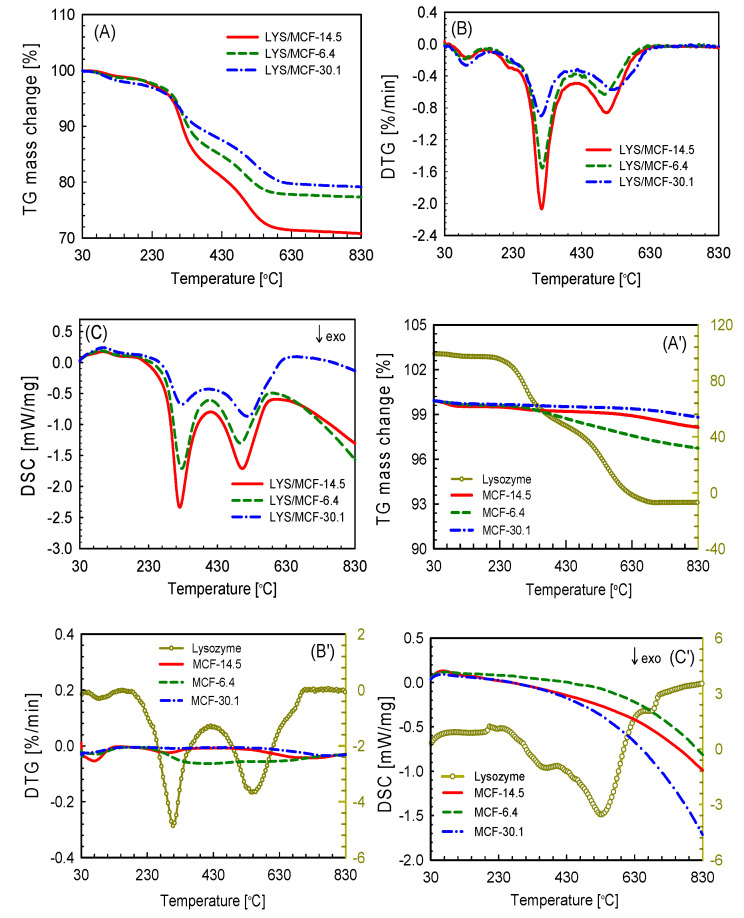
Thermogravimetric analyzer (TG) profiles in synthetic air for selected LYS/MCF composites (**A**), native protein and pure MCF supports (**A**’); DTG curves in synthetic air for selected LYS/MCF composites (**B**), native lysozyme and pure MCF supports (**B**’); DSC curves for biocomposites (**C**), pure lysozyme and MCF silica supports (**C**’).

TG/DTG/DSC curves of thermal decomposition of native lysozyme, pure silica curriers, and silica/LYS composites in the air are presented in Figure 4A,A’–C,C’. The mass losses (TG), the corresponding derivatives (DTG), and differential scanning calorimetry values (DSC) for MCF-14.5, MCF-6.4, MCF-30.1 without and with immobilized protein analyzed in oxidation atmosphere are gathered in Table 5 and Table 6. Basing on the presented results one can see that pure MCF materials decompose in one main step under oxidative conditions. The total mass losses in the range 185 °C to 830 °C estimated for MCF-14.5, MCF-6.4, MCF-30.1 materials were about 1.57%, 3.09% and 1.03%, respectively (Figure 4A’). The LYS/MCF composites are thermally stable up to the temperature ~165 °C in the atmosphere of synthetic air. Afterwards, the decomposition of the LYS/MCF composites runs as two major stages in the temperature range 165–420–830 °C with *T_max*1*_* (the maximum temperatures of mass loss in first decomposition step) and *T_max*2*_* (the maximum temperatures of mass loss in second decomposition step) given in Table 5. It should also be noted that for LYS/MCF composites, the weight loss (1.2 to 2%) in initial decomposition temperature (*M_loss,IDT_*) 30–165 °C is attributed to the removal of water (endothermic process, Figure 4A, Table 5). In turn, the mass loss in the first main step (*M_loss*1*_*) at temperatures 165–420 °C is related to the removal of weakly bound albumin from the silica supports (van der Waals forces) and in the second one (*M_loss*2*_*) above 420 °C for LYS/MCF composites are associated to the removal of protein molecules strongly linked with the silica materials (electrostatic interactions) [39,40]. TG/DTG analysis (Figure 4A,B and Table 5) for MCF-14.5/LYS composite shows that the first main decomposition step appears in the temperature range 165–420 °C with *T_max*1*_* ~323 °C, and with the mass loss 17.3%. The second step of decomposition is visible in the temperature range 420–830 °C with the mass loss of 10.8% occurred in *T_max*2*_* ~508 °C. Considerably smaller mass losses 13.4%, 9.9% (*M_loss*1*_*), and 8.9%, 8% (*M_loss*2*_*) were obtained for LYS/MCF-6.4, LYS/MCF-30.1 composites, respectively. For MCF-6.4 silica with a small pore size the first step of decomposition occurs with *T_max*1*_* ~327 °C and in the second step with *T_max*2*_* ~498 °C. Likewise, in the case of MCF-30.1 sample with wider pores the first and second stage of decomposition is found at *T_max*1*_* ~321 °C and *T_max*2*_* ~520 °C (Table 5). These decomposition stages are directly connected to the presence of LYS in the studied materials. These results significantly confirm that increasing the adsorption of lysozyme on MCF leads to greater weight loss during the heating process of the studied biomaterials. This effect is greater for MCF-14.5 material with a pore diameter of about 3.5 times larger than the hydrodynamic diameter of protein (4 nm). It may be concluded that the total mass loss (*M_loss,TOTAL_*) for LYS/MCF-14.5 composite is ca. 5.7% higher than for LYS/MCF-6.4, and almost 9.4% greater in comparison to LYS/MCF-30.1.

Additionally, the DSC curves (Figure 4C) for three studied MCF/protein composites reveal two exothermic peaks in the region of high temperatures. The presence of the first exothermic peak situated in the range 270–373 °C with *T_peak_* ca. 321–328 °C, and the second exothermic peak in the range 447–620 °C with *T_peak_* ca. 498–519 °C (Table 6) is directly connected with the main decomposition step of LYS, which corresponds to the TG mass losses. Also, the characteristic temperatures (*T_onset_,* the temperature of decomposition initiation; *T_peak_,* the maximum decomposition temperature; *T_end_,* the final decomposition temperature) obtained from DSC (Figure 4C and Table 6) are comparable to those obtained from TG analysis.

**Figure 5:**
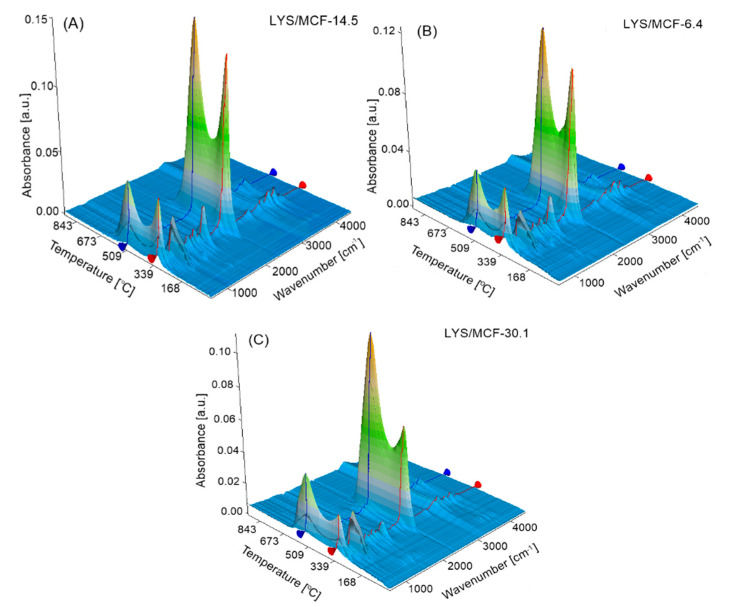
3D FTIR spectrum of (**A**) LYS/MCF-14.5, (**B**) LYS/MCF-6.4, and (**C**) LYSMCF-30.1 composites registered at two peaks temperatures *T_peak*1*_* (red line) and *T_peak*2*_* (blue line) marked from DSC curves.

**Figure 6:**
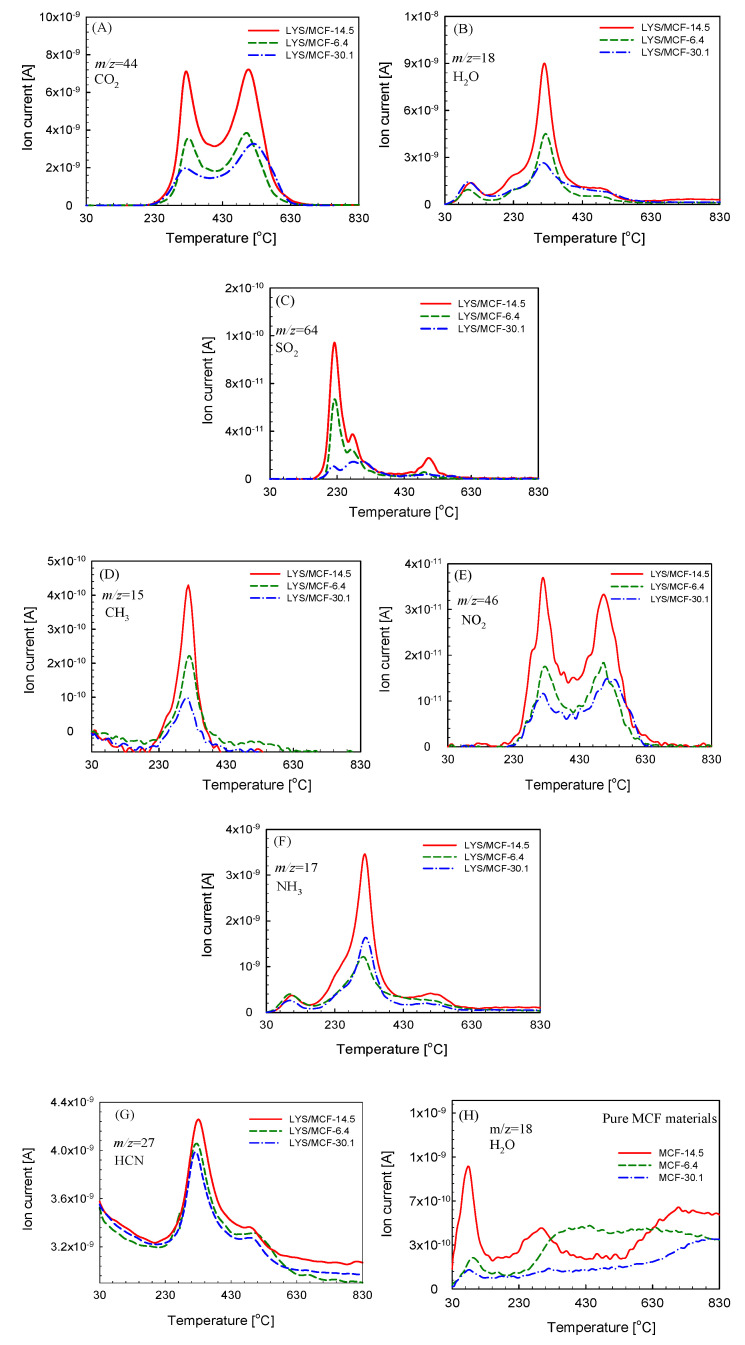
QMS profile of gaseous products emitted during decomposition of three selected LYS/MCF-14.5, LYS/MCF-6.4, LYS/MCF-30.1 composites vs. temperature at *m/z*: (**A**) 15 (CO_2_); (**B**) 18 (H_2_O); (**C**) 64 (SO_2_); (**D**) 15 (CH_3_); (**E**) 46 (NO_2_); (**F**) 17 (NH_3_); (**G**) 27 (HCN) in air atmosphere; (**H**) MS profile of H_2_O vs. temperature for pure MCF-14.5, MCF-6.4 and MCF-30.1 supports.

To explain the thermal decomposition routes, as well as its mechanisms for the studied biomaterials, the analysis of the gaseous products emitted during their decomposition was conducted by TG/FTIR and TG/QMS technique. 3D FTIR spectrum of the studied LYS/MCF composites gathered at the temperatures corresponding to the main thermal degradation stages on the DSC curves (*T_peak*1*_* and *T_peak*2*_*) in the air atmosphere are presented in Figure 5A–C. The typical QMS spectra of the characteristic ion currents (*m/z*) of gaseous products formed during the decomposition of three selected MCF supports with immobilized lysozyme obtained in the air are shown in Figure 6A–G.

Figure 6H presents QMS profiles of the main product, H_2_O, formed during the decomposition of pure MCF-14.5, MCF-6.4, MCF-30.1 materials. According to these results for all native silica materials, the ion current signal at *m/z* = 18 is observed which is characteristic for water forming as the main decomposition product (Figure 6H). It is confirmed by the FTIR spectra in the ranges 3570 cm^−1^ to 3900 cm^−1^, 1390 cm^−1^ and 1870 cm^−1^ corresponding to rotation/vibration transition within water molecules in the vapor phase—stretching vibration and symmetric bending.

TG/QMS/FTIR results significantly indicate the formation of various gaseous products during the decomposition process of three studied biocomposites. At the main thermal degradation stages at *T_peak*1*_* and *T_peak*2*_*, the absorption bands at 2310 cm^−1^, 2360 cm^−1^ and 669 cm^−1^ characteristics for carbon dioxide (Figure 5A–C) are observed. Two first bands correspond to R and P-branches of asymmetric stretching vibration and the third band is connected with symmetric bending vibrations of CO_2_. The presence of this compound is also evidenced in the MS spectra of the characteristic ion current at *m/z* = 44 (Figure 6A). Moreover, the absorption bands at 3735 cm^−1^, 3503 cm^−1^, 1756 cm^−1^ correspond to the O-H stretching vibrations of water molecules in the vapor phase (both stretching vibrations and symmetric bending) (Figure 5). The MS signal corresponding to *m/z* = 18 confirms the appearance of water vapor in the mixture of gases evolved during thermal decomposition of protein/silica composites (Figure 6B). Among the decomposition products, one can find also the signals recorded for SO_2_ (at *m/z* = 64) (Figure 6C) which comes from the sulfonate groups in biocomposites. It is also confirmed by the FTIR absorption bands at ca. 1370 cm^−1^ (asymmetric stretching vibration of the SO_2_ molecule). In this decomposition stage, the FTIR absorption bands at 2896 cm^−1^ and 2972 cm^−1^ (Figure 5) are found which may be attributed to the CH stretching vibrations characteristic for the aliphatic groups appearing in the gaseous decomposition products of protein. They give the MS signal *m/z* = 15 (Figure 6D) corresponding to methyl groups CH_3_. Moreover, the FTIR absorption band at ca. 1617 cm^−1^ is also identified. It corresponds to the asymmetric stretch of NO_2_ (the signal at *m/z* = 46 on the MS spectrum, Figure 6E). Additionally, among the products observed during the thermal decomposition NH_3_ presence at *m/z* = 17 (Figure 6F) is detected and proved by FTIR absorption bands of medium intensities at 930 cm^−1^ and 965 cm^−1^ (maxima of rotational branches of the symmetric bending vibration), and 1626 cm^−1^ (the Q-branch of asymmetric bending vibration) (Figure 5). Moreover, the presence of HCN in the gaseous phase is found on the MS spectrum at *m/z* = 27, and on the FTIR absorption band at 714 cm^−1^ (Figure 5 and Figure 6G).

It is worth noting that for LYS/MCF-14.5 the QMS spectra show the higher ion currents (*m/z =* 15, 17, 18, 27, 44, 46, 64) of gaseous products formed during the decomposition of protein/silica material: CH_3_, NH_3_, H_2_O, HCN, CO_2_, NO_2_, SO_2_ in comparison to LYS/MCF-6.4 and LYS/MCF-30.1. It confirms that more protein molecules are immobilized on the MCF-14.5 support. The same conclusions are supported by the analysis of TG/FTIR spectra for the studied biomaterials by higher intensities of absorption bands. It may be concluded that this effect decreases in the series: MCF-14.5 > MCF-6.4 > MCF-30.1. These results are in good agreement with the N_2_ adsorption/desorption analysis and batch adsorption experiment.

### 2.5. Microscopic Analysis (SEM, TEM)

**Figure 7:**
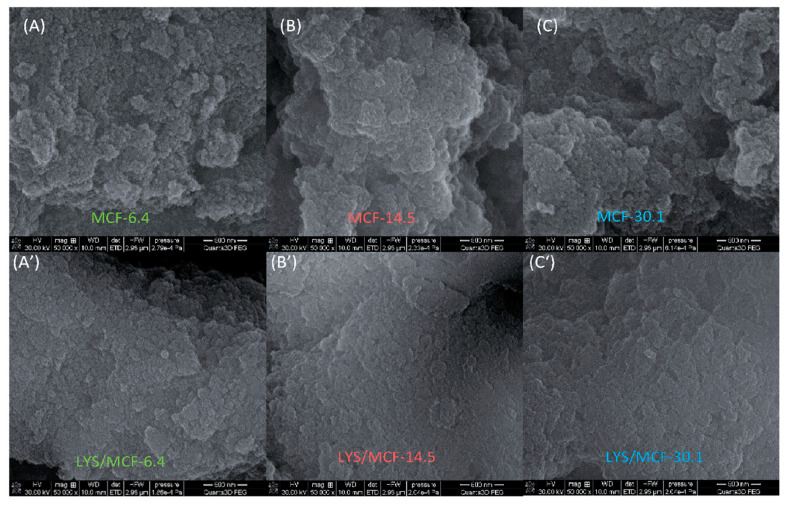
SEM micrographs of MCF-6.4 (**A**, magnification 50,000), MCF-14.5 silica (**B**, magnification 50,000), and MCF-30.1 (**C**, magnification 50,000) and after protein adsorption (**A**’, **B**’, **C**’, magnification 50,000), respectively.

**Figure 8:**
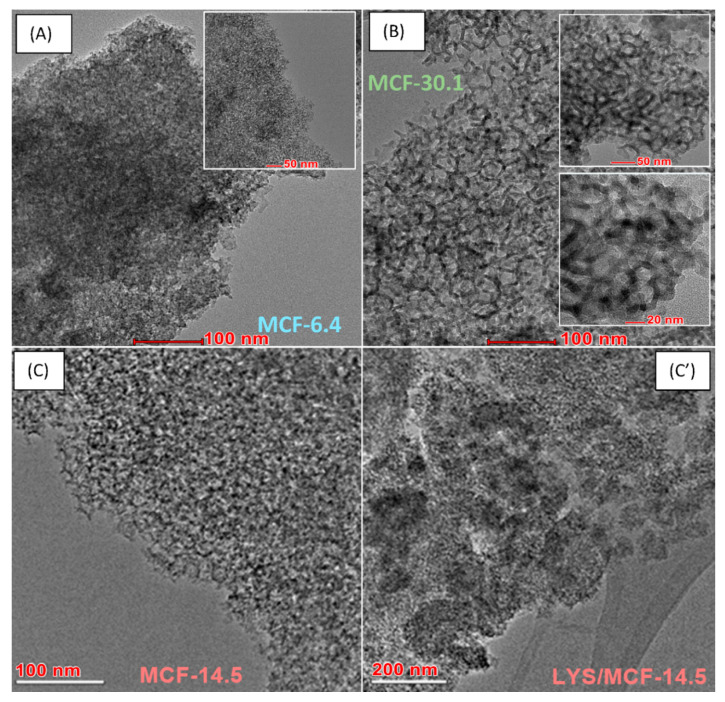
(**A**) HRTEM-BF micrographs showing the surface morphology and nanostructure of the MCF-6.4 (**A**), MCF-30.1 (**B**), and MCF-14.5 support before (**C**), and (**C**’) after immobilization of LYS molecules.

The scanning electron microscopy (SEM) and the high-resolution transmission electron microscopy HRTEM-BF (bright field) allow comparing the surface morphology, texture, and micro-nanostructure of the native MCF supports with different pore sizes and protein/silica composites. The two-dimensional (2D) micro-nano surface morphology visualization of the pure MCF-6.4, MCF-14.5, MCF-30.1 supports, and the biocomposites registered by SEM are presented in Figure 7(A–C) and Figure 7(A’–C’), respectively. As can be seen, the lysozyme immobilization on the silica support with different pore sizes changes the grain surface morphology. In the case of all biocomposites the surface becomes smoother and more uniform comparing with the pure silica material [41]. Moreover, one can observe that the surface roughness changes as follows: MCF-6.4 > MCF-14.5~MCF-30.1. This observation confirms the changes in adsorption density obtained from the batch adsorption experiment (see Figure 1D). Figure 8A–C shows a comparison of TEM texture/topography micrographs at nano-level of pure MCF-6.4, MCF-30.1, and MCF-14.5 silica support as well as LYS/MCF-14.5 composite (Figure 8C’). It can be seen that HRTEM-BF micrographs for pure MCF-14.5, MCF-6.4, and MCF-30.1 silica materials confirm a porous structure of studied materials with regular 3D pore system with large spherical cells interconnected by narrow interconnectivities. The pore diameter estimated from HRTEM micrographs is ~15 nm for MCF-14.5, ~7 nm for MCF-6.4 and ~31 nm for MCF-30.1, which is consistent with N_2_ sorption parameters. Moreover, the TEM micrographs show the immobilized lysozyme (as a circle-spiral form) on the surface of the MCF-14.5 support (Figure 8C’) [41].

## 3. Materials and Methods

### 3.1. Reagents

Lysozyme from chicken egg white (lyophilized powder, protein ≥90%, ≥40,000 units/mg protein Cat. No. L6876) and 0.1 M phosphate buffer saline pH = 7.4, (BioPerformance Certified Buffers, Cat. No. P5368) were obtained from Sigma-Aldrich (Oakville, ON, Canada). The non-ionic triblock copolymers Pluronic PE9400 (EO_21_PO_47_EO_21_, M_av_ = 4600) from BASF (Baden Aniline and Soda Factory, Warsaw, Poland) and Pluronic P123 (EO_20_PO_70_EO_20_, M_w_ = 5800) from Sigma-Aldrich (Oakville, ON, Canada) were used as structure-directing agents. Tetraethylorthosilicate used as a silica source (TEOS 98%), 1,3,5-trimethylbenzene (TMB) as a pore expanding agent, ammonium fluoride, (NH_4_F ≥98.0%) as a tuning window pore size-mineralizing agent, hydrochloric acid (37%) and other reagents of analytical grade were purchased from Sigma-Aldrich (Oakville, ON, Canada) and used without further purification. Water was purified using a Millipore apparatus (resistivity of 18.2 MΩ·cm at 25 °C).

Lysozyme with its well-determined structure is a small molecular protein (14.3 kDa) with a hydrodynamic radius of ~2 nm and is composed of a single polypeptide chain of 129 amino acids in the form of five α-helices, three antiparallel β-sheets and a large number of random coils and β-turns [42,43]. It has a prolate spheroid shape with two characteristic cross-sections: a side of approximate dimensions 4.5 × 3.0 nm^2^ (shape similar to an ellipsoid) and an end of dimensions 3.0 × 3.0 nm^2^ [44]. The isoelectric point for lysozyme is pH_IEP_~11, at pH = 7 it is positively charged [45,46]. This enzyme has high structural stability and inflexibility within pH wide range 1.5 to 12 because the rigidity of the single-chained LYS molecule is imposed by four internal disulphide bonds that help maintain its tertiary structure [47]. This globular protein belongs to the family of hydrolases which, thanks to its biological function (the hydrolysis of the polysaccharide forming the bacterial cell wall), can be applied as an antimicrobial, anti-inflammatory, antiviral, antiseptic, antitumor agents [48].

### 3.2. Synthesis of Mesocellular Foam

Mesoporous MCF silica adsorbents with different pore structures were synthesized according to a previously published procedure [49,50] with some modifications described in the papers [51,52]. In the preparation of pure MCF support, Pluronic (P123 or PE9400) (4 g) was dissolved in 180 mL of 1.6 M aqueous HCl solution and stirred over 2 h at room temperature. Then, the established amount of TMB (polymer/TMB mass ratios were varied) was introduced to the mixture, and the resulting liquid was heated to 40 °C under continuous vigorous stirring by using a digitally controlled mechanical stirrer (250 rpm, 45 min). Then, the desired amounts of TEOS (the mass proportions of polymer/TEOS were changed) was introduced to the reaction mixture, and it was stirred for another 20 h at the same temperature. The resulting solution was transferred to an autoclave and aged at elevated temperatures (110–120 °C) for 24–144 h. In the case of some samples, 46 mg of NH_4_F in 0.3 mL H_2_O was added if desired as the mineralizing agent to increase the window pore size. Finally, the synthesized precipitate was thoroughly washed with distilled water and dried in air. Detailed synthesis conditions of MCF samples are given in Table S2 (Supplementary Material).

### 3.3. Adsorption Experiment

#### 3.3.1. Adsorption Equilibrium

The equilibrium studies of LYS adsorption on the series of MCF adsorbents with varied porosity were performed by using the static methods. Before the experiment, the MCF material was dried at 150 °C. A series of stock protein solutions at different concentrations (1–5 mg/mL) was prepared by dissolving appropriate amounts of protein in phosphate-buffered saline solutions at pH = 7.4. Then, 100 mg of pure MCF materials were contacted with the protein solution. The Erlenmeyer flasks with resulting suspensions were placed in the incubator shaker (New Brunswick Scientific Innova 40R Model, Eppendorf AG, Hamburg, Germany) and stirred at 25 °C at 110 rpm speed until equilibrium was reached (24 h). After attaining equilibrium the equilibrium concentrations of proteins were determined using UV–Vis spectrophotometer Cary 100 (Varian Inc., Melbourne, Australia) at the wavelength of 281 nm. The adsorbed amount of protein was calculated from the mass balance equation:

(1) $$a_{eq}=\frac{\left(c_{o}-c_{eq}\right)\cdot V}{w}$$

where: *a_eq_* is the equilibrium adsorbed amount [mg/g], *c_o_* is the initial concentration of protein solution [mg/mL], *c_eq_* is the protein equilibrium concentration [mg/mL], *V* is the solution volume [ml], and *w* is the MCF mass [mg].

The experimental protein adsorption isotherms from aqueous solutions were analyzed by applying the Generalized Langmuir (GL) isotherm equation [53]:

(2) $$a_{eq}/a_{m}={\left[\frac{{\left(Kc_{eq}\right)}^{m}}{1+{\left(Kc_{eq}\right)}^{m}}\right]}^{m/n}$$

where: *a_m_* is the adsorption capacity; *m*, *n* is the heterogeneity parameters (0 < *m, n* ≤ 1) describing the shape (asymmetry) of adsorption energy distribution function; *K* is the equilibrium constant related to characteristic adsorption energy.

GL equation is applied for the analysis of the localized physical adsorption on energetically heterogeneous solids. For the specific values of *m* and *n* parameters GL equation changes form into 4 simpler isotherms equations: Langmuir (L) (GL: *m* = *n* = 1); Langmuir-Freundlich (LF) (GL: 0 < *m* = *n* ≤ 1); Generalized Freundlich (GF) (GL: *n* = 1, 0 < *m* ≤ 1); Tóth (T) (GL: *m* = 1, 0 < *n* ≤ 1).

#### 3.3.2. Adsorption Kinetics

The adsorption kinetic measurements were performed by means of the UV-Vis spectrophotometer Cary 100 (Varian Inc., Melbourne, Australia) with a quartz flow cell to analyze a solute concentration in a closed system [54,55,56]. The LYS solution (200 mL) with an established initial concentrate on of 0.4 mg/mL was conducted with a known amount of mesoporous silica (250 mg) in a thermostated vessel at constant temperature (25 °C). The suspension was stirred during the experiment by applying a digitally controlled mechanical stirrer (110 rpm). At definite time intervals the sample of protein solution was collected automatically to the flow cell of the spectrophotometer and the absorbance UV spectra in the wavelength range 200–400 nm were collected. Afterwards, the protein solution was returned to the reaction vessel. Finally, the concentration vs. time and the adsorption vs. time profiles for the protein adsorption system were calculated from the obtained spectra.

The measured kinetic data were analyzed by using the multi-exponential (m-exp) equation [30]:

(3) $$c=\left(c_{0}-c_{eq}\right)\sum_{i=1}^{n}f_{i}exp\left(-k_{i}t\right)+c_{eq}$$

where: *c*—the actual adsorbate concentration, *k_i_*—the rate constant (*i* = 1, 2, ..., *n*), *f_i_*—the coefficient determining a fraction of a solute adsorbed with rate *k_i_*. Moreover, the adsorption kinetic half-time was estimated as a time needed for attaining ½ of concentration change.

The kinetic experimental data were also studied by applying the other kinetic equations and models: first-order (FOE), second-order (SOE), mixed-order (MOE), fractal first-order (f-FOE), fractal second-order (f-SOE), fractal mixed-order (f-MOE) equations, and the intraparticle diffusion (IDM, Crank) and pore diffusion (PDM, McKay) models (Supplementary Material) [30]. The non-linear LSQ optimization was applied in the calculations taking into account the difference between the experimental and fitted adsorbate concentrations.

### 3.4. Potentiometric Titration

The surface acid-base character (surface charge and pH of zero charge—*pH_pzc_*) of the pure MCF materials and MCF with the immobilized LYS were determined by potentiometric titration. The measurements were carried out in the thermostatic vessel at 25 °C applying a Dosimat 765 automatic burette (Metrohm, Herisau, Switzerland) connected with a precision pH-meter (PHM 240, Radiometer, Copenhagen, Denmark). The details of the potentiometric titration measurements for pure MCF sorbents were as follows: 30 mL of the stock electrolyte (0.1 M NaCl) was acidified with 0.3 mL of 0.5 M HCl solution and transferred into a thermostatic quartz vessel. To prevent contamination with CO_2_, the pure nitrogen flow was used throughout the titration (1–2 bubbles per second). The determined amounts of MCF samples (0.1 g) were added into the electrolyte solution and then the resulting suspension of adsorbent was titrated by using 0.2 M NaOH recording pH changes.

The potentiometric titration experiment for MCF adsorbents with immobilized lysozyme was carried out as follows: the MCF samples (0.1 g) were added to a thermostatic vessel containing 30 mL of lysozyme solution of concentration 5 mg/mL in 0.1 M NaCl. Then, the system was acidified (0.3 mL of 0.5 M HCl solution), and the adsorption process was carried out at 25 °C for 24 h and finally the resulting mixture protein/sorbent suspension was also titrated using 0.2 M NaOH.

The obtained potentiometric titration curves (pH solution vs. NaOH volume) for MCF materials and LYS/MCF systems were converted into the surface charge density curves, using the equation [57]:

(4) $$q_{s}=\frac{F\cdot \Delta n_{H^{+}}}{S_{BET}}$$

where: *q_s_*—the surface charge, $\Delta n_{H+}$ —the ion mole number for 1 g of material, *F*—the Faraday’s constant, *S_BET_*—the specific surface area of adsorbents.

### 3.5. Nitrogen Adsorption/Desorption Isotherms

The porous structure evaluation of mesoporous silica before and after lysozyme adsorption were thoroughly characterized by using a low-temperature adsorption/desorption of nitrogen at −196 °C applying the automatic ASAP 2020 sorption analyzer (Micromeritics Instrument Corp., Norcross, GA, USA). Prior to the measurement, the pure MCF supports and LYS/MCF composites were outgassed (4 µmHg), respectively, at 150 °C and 30 °C for 24 h in degas port of analyzer. The obtained adsorption/desorption isotherms were used to evaluate the porous structure of the studied MCF adsorbents as well as protein/silica composites. The specific surface area *(S_BET_)* was calculated using the Brunauer-Emmett-Teller (BET) equation. Meanwhile, the total pore volumes *(V_t_)* were estimated from the single point adsorption value at the relative pressure (*p/p_o_*) of 0.99. To assess the values of the external surface area (*S_ext_*) and the mesopore volume (*V_mes_*) the *α_s_* plot was used [58]. Calculation of the pore size distributions (PSD) were determined using the adsorption and desorption branches of the isotherms by means of the Barrett–Joyner–Halenda (BJH) procedure for cylinder pores [59]. The pore diameters were derived from the PSD maxima (mode, *D_mo_*) and PSD average (*D_av_*). In turn, the mean hydraulic pore diameters were calculated from the BET surface areas and total pore volumes *D_h_* = 4·*V_t_*/*S_BET_*.

### 3.6. Scanning Electron Microscopy (SEM) and Transmission Electron Microscopy (TEM)

The surface morphology, topography, and structure at the micro and nano level of silica support without and with adsorbed protein was examined by using the scanning electron microscope (SEM) Quanta 3D FEG (FEI, Field Electron and Ion Co., Hillsboro, OR, USA) and the high-resolution transmission electron microscope S/TEM Titan3TM G2 60-300 (FEI Company, Hillsboro, OR, USA) equipped with a field-emission electron gun (FEG) in high-resolution bright-field imaging (HRTEM-BF) in TEM mode.

### 3.7. Thermal Analysis Coupled with FTIR and MS

The thermal analysis was used to examine the thermal stability, behavior, and decomposition of the MCF materials before and after LYS adsorption, as well as to determine the presence of protein on the silica surface or in the pores. The thermal experiment was carried out on the apparatus STA 449 Jupiter F1 (Netzsch, Selb, Germany). The samples (~18 mg) in aluminum crucibles were heated in the temperature range 30–950 °C with a heating rate of 10 °C/min under a dynamic atmosphere of synthetic air with a flow rate of 50 mL/min. The sensor thermocouple type S TG-DSC and empty Al_2_O_3_ crucible as a reference were used. The identification of gas products emitted during decomposition of the studied materials was detected and analyzed by quadrupole mass spectrometer QMS 403C Aëolos (Netzsch, Selb, Germany) and Fourier transform infrared spectroscopy FTIR spectrometer Brucker (Ettlingen, Germany) coupling on-line to STA instrument. The QMS data were collected in the range of 10 to 300 amu. The FTIR spectra in the spectral range 600–4000 cm^−1^ with 16 scans per spectrum at a resolution 4 cm^−1^ were recorded.

## 4. Conclusions

The synthesized series of mesoporous MCF silica supports differentiated by pore diameters (6–30 nm) and surface areas (250–720 m^2^/g) were applied for lysozyme immobilization by physical adsorption. For the first time, special attention was paid to the correlation between the MCF pore size and specific surface area, and lysozyme adsorption capacity and rate. The obtained results allow to optimize/design the structural characteristics of the solid support with respect to biomolecule adsorption and surface density for potential biomedical/biophysical applications. We found that the strongest adsorption was obtained for MCF-14.5 with the medium pore size which might be explained by the optimum relation of lysozyme molecular size and support pore diameter responsible for the increase of the adsorption forces. In the case of other materials with lower and higher pore sizes, the protein adsorbed amount decreased proportionally to a relation: pore diameter/protein diameter. The lysozyme adsorption process was the slowest for MCF-6.4 with the narrowest pores due to the hindered diffusion of protein molecules into the support pores. However, it is the quickest for MCF-30.1 with the largest pores facilitating the diffusion and adsorption. In the case of MCF-14.5, the adsorption process is slightly slower in comparison to MCF-30.1 which means that the pore sizes of this material do not disturb protein diffusion. The kinetic profiles for MCF-30.1 and MCF-6.4 achieved the comparable close to equilibrium adsorption values, however, for MCF-14.5 the equilibrium adsorption was higher. The changes in pore characteristics after lysozyme adsorption revealed that the protein molecules were adsorbed inside the channels of mesoporous adsorbents as well as located on the surface. Considering the positive charge of lysozyme at pH = 7.4 and the negative charge of silica, one can assume that the electrostatic attractive interactions play a significant role in the immobilization process. After protein adsorption pH*_pzc_* for biomaterials stabilizes. The microscopic analyses revealed that topography, texture, and micro/nanostructure of the biocomposite surface were completely different from that of pure silica. The morphology and texture of the biocomposite surface were less porous, more homogeneous, and flat. The differences in protein adsorption and surface density for the biocomposites of various pore sizes are evident.

In further research, we will focus on estimating the LYS activity after adsorption on the mesoporous support and on studying the possible pore size effect to design a protein-containing biocomposite with better stability/activity for attaining effective mesoporous support for enzyme delivery and potential biotechnological applications [60].

## Figures and Tables

**Table 1:** Textural parameters of the obtained mesoporous silica materials.

Material	Surface Area [m^2^/g]		Pore Volume [cm^3^/g]		Pore Diameter by BJH Adsorption		Pore Diameter by BJH Desorption		Pore Size (Average Hydraulic) [nm]
					PSD Average [nm]	Mode [nm]	PSD Average [nm]	Mode [nm]	
	^a^ *S_BET_*	^b^ *S_ext_*	^c^ *V_t_*	^d^ *V_mes_*	^e^ *D_av, ads._*	^f^ *D_mo, ads._*	^g^ *D_av,des._*	^h^ *D_mo,des._*	^i^ *D_h_*
MCF-6.4	716	29	1.15	1.09	5.8	6.6	5.2	4.9	6.4
MCF-7.4	685	38	1.26	1.18	7.2	7.9	6.5	6.4	7.4
MCF-8.7	522	63	1.14	1.03	8.3	8.9	7.4	8.3	8.7
MCF-10.1	533	72	1.32	1.19	9.9	10.1	8.8	9.3	10.1
MCF-12.4	569	65	1.76	1.63	13.4	13.7	11.9	10.8	12.4
MCF-14.5	547	135	1.98	1.74	16.5	16.6	13.6	12.6	14.5
MCF-15.5	494	262	1.91	1.44	20.1	17.0	16.9	17.9	15.5
MCF-20.3	435	41	2.21	2.00	25.2	27.7	20.6	22.2	20.3
MCF-25.8	301	231	1.94	1.45	32.3	27.9	23.3	21.8	25.8
MCF-27.7	267	67	1.85	1.73	34.5	38.0	26.8	29.6	27.7
MCF-30.1	250	61	1.88	1.76	37.5	38.7	30.2	30.9	30.1

^a^ S*_BET_*, BET specific surface area; ^b^ S*_ext_*, external surface area; ^c^ V*_t_*, total pore volume; ^d^ V*_mes_*, mesopore volume; ^e,g^ D*_av_*, *_ads.,des_*., BJH average pore diameters for cylinder pores geometry from adsorption and desorption branch of isotherms; ^f,h^ D*_mo_*_, *ads., des*.,_ pore diameters from the PSD maxima by BJH (mode) obtained from adsorption/desorption isotherms for cylinder pores geometry; ^i^ D*_h_*_,_ average hydraulic pore diameter.

**Table 2:** Parameters of the Generalized Langmuir (GL) isotherm equation (optimized to (LF) Langmuir−Freundlich or (L) Langmuir Isotherms) characterizing the adsorption systems investigated.

Adsorption System	Fitted Isotherm	*a_m_*	*m*	*n*	log *K*	*R^2^*	SD*(a)*
LYS/MCF-14.5	LF	0.82	0.40	0.40	0.25	0.997	0.008
LYS/MCF-6.4	GL	0.37	0.50	1	−0.32	0.997	0.004
LYS/MCF-30.1	L	0.29	1	1	0.71	0.985	0.006

*a_m_*_,_ adsorption capacity; *m*, *n,* heterogeneity parameters describing the shape (asymmetry) of adsorption energy distribution function; *K*, equilibrium constant related to characteristic adsorption energy; *R_2_,* determination coefficients; SD, standard deviations.

**Table 3:** Optimized parameters of m-exp eq.

System	*f*_1_, log*k*_1_	*f*_2_, log*k*_2_	*f*_3_, log*k*_3_	*t_1/2_* [min]	SD(c)/c_o_ [%]	1-R^2^
LYS/MCF-6.4	0.054, −2.24	0.946, −3.91	0	358.7	0.337	0.0037
LYS/MCF-14.5	0.231, 0.21	0.125, −2.48	0.644, −9.99	1.7	0.414	0.0027
LYS/MCF-30.1	0.187, 0.55	0.813, −4.02	0	0.5	0.723	0.021

*f_i_* (*i* = 1, 2, ..., *n*), coefficients determining a fraction of adsorbate adsorbed with the rate constant *k_i_*; log*k_i_*, rate constant logarithms; *t_1/2_*, adsorption half-time; SD(c/c_o_), standard deviation; 1-R^2^, indetermination coefficient.

**Table 4:** Pore structure parameters of the investigated pure supports and biocomposites calculated from N_2_ adsorption/desorption isotherms. Adsorption conditions: *c_0_* = 5 mg/mL; *t_ads_* = 24 h; T = 25 ˚C; pH = 7.4.

Material	Surface Area [m^2^/g]		Pore Volume [cm^3^/g]		Pore Diameter by BJH Adsorption		Pore Diameter by BJH Desorption		Pore Size (Average Hydraulic) [nm]
					PSD Average [nm]	Mode [nm]	PSD Average [nm]	Mode [nm]	
	^a^ *S_BET_*	^b^ *S_ext_*	^c^ *V_t_*	^d^ *V_mes_*	^e^ *D_av, ads._*	^f^ *D_mo, ads._*	^g^ *D_av,des._*	^h^ *D_mo,des._*	^i^ *D_h_*
MCF-6.4	716	29	1.15	1.09	5.8	6.6	5.2	4.9	6.4
MCF-14.5	547	135	1.98	1.74	16.5	16.6	13.6	12.6	14.5
MCF-30.1	250	61	1.88	1.76	37.5	38.7	30.2	30.9	30.1
LYS/MCF-6.4	346	7	0.49	0.48	5.2	6.3	4.7	5.5	5.7
LYS/MCF-14.5	38	22	0.12	0.08	12.5	12.2	10.9	10.7	12.6
LYS/MCF-30.1	114	25	0.50	0.44	18.5	20.7	14.9	17.6	17.5

^a^S *_BET_*, BET specific surface area; ^b^ S*_ext_*, external surface area; ^c^ V*_t_*, total pore volume; ^d^ V*_mes_*, mesopore volume; ^e,g^ D*_av_*, *_ads.,des_*., BJH average pore diameters for cylinder pores geometry from adsorption and desorption branch of isotherms; ^f,h^ D*_mo,ads.,des.,_* pore diameters from the PSD maxima by BJH (mode) obtained from adsorption/desorption isotherms for cylinder pores geometry; ^i^ D*_h_*, average hydraulic pore diameter.

**Table 5:** TG and DTG data obtained in air atmosphere for LYS/MCF composites.

Material	TG				DTG	
	***M**_loss,IDT_* [%]	***M**_loss*1*_* [%]	***M**_loss*2*_* [%]	***M**_loss,TOTAL_* [%]	***T**_max*1*_* [°C]	***T**_max*2*_* [°C]
	30–165 [°C]	165–420 [°C]	420–830 [°C]			
LYS/MCF-14.5	1.2	17.3	10.8	29.3	323	508
LYS/MCF-6.4	1.3	13.4	8.9	23.6	327	498
LYS/MCF-30.1	2.0	9.9	8.0	19.9	321	520

*M_loss,IDT_*, mass loss at initial decomposition temperature; *M_loss*1,2*_*, mass loss in main (first or second) decomposition step *M_loss,TOTAL_*, total mass loss; *T_max*1,2*_*, maximum temperatures of mass loss in first or second decomposition step.

**Table 6:** DSC data obtained in synthetic air for MCF materials before and after lysozyme adsorption.

Material	DSC							
	*T_onset1_* [°C]	*T_peak1_* [°C]	*T_end1_* [°C]	*∆H* [J/g]	*T_onset2_* [°C]	*T_peak2_* [°C]	*T_end2_* [°C]	*∆H* [J/g]
LYS/MCF-14.5	283	321	366	−654	448	503	587	−452	
LYS/MCF-6.4	282	327	372	−491	447	498	588	−338	
LYS/MCF-30.1	270	328	373	−197	455	519	620	−408	

*T_onset_,* temperature of the decomposition initiation; *T_peak_*, maximum decomposition temperature; *T_end_*, final decomposition temperature; *∆H,* the heat generated during the decomposition process obtained by the integration of the thermal peaks.

## Supplementary Materials

Supplementary Materials can be found at https://www.mdpi.com/1422-0067/21/15/5479/s1. Figure S1. (A) Comparison of nitrogen adsorption/desorption isotherms for selected MCF materials with different pore sizes (MCF-6.4 nm, MCF-14.5 nm, MCF-30.1 nm). (B, C) Pore size distributions calculated by using BJH method for the adsorption and desorption branches of isotherms. Figure S2. (A) Comparison of N2 adsorption/desorption isotherms before and after LYS adsorption for MCF-14.5, and MCF-6.4, MCF-30.1 (inset plots). (B) Differential pore size distributions (PSDs) evaluated from the BJH model based on desorption data for pure MCF-14.5 support and covered by the LYS molecules. Inset is the pore size distributions for pure MCF-6.4, MCF-30.1 supports, and after LYS adsorption. Figure S3. Influence of hydraulic pore diameter (*D_h_*) and surface area (*S_BET_*) on the point of zero charge (pH_pzc_) of MCF materials after LYS adsorption. Inset: variations pH_pzc_ as a function of *D_h_* and *S_BET_* for pure supports. Table S1. Relative standard deviations SD(c)/co for m-exp, FOE, SOE, MOE, f-FOE, f-SOE, F-MOE, McKay pore diffusion (PDM) and IDM model (Crank). Table S2. Preparation conditions in MCF synthesis.
