# Supplementary material for: Mesocellular Silica Foams (MCFs) with Tunable Pore Size as a Support for Lysozyme Immobilization: Adsorption Equilibrium and Kinetics, Biocomposite Properties
Source: Int J Mol Sci. 2020 Jul 31;21(15):5479. doi: 10.3390/ijms21155479 (PMC7432670; doi:10.3390/ijms21155479)
Supplement: Supplementary file 1 [file ijms-21-05479-s001.pdf]

Supplementary material for the paper:

Mesocellular Silica Foams (MCFs) with Tunable Pore Size as a Support for Lysozyme Immobilization: Adsorption Equilibrium and Kinetics, Biocomposite Properties

Agnieszka Chrzanowska*, Anna Derylo-Marczewska and Malgorzata Wasilewska

Department of Physical Chemistry, Institute of Chemical Sciences, Faculty of Chemistry, Maria Curie-Sklodowska University in Lublin, M. Curie-Sklodowska Sq. 3, 20-031 Lublin, Poland; annad@hektor.umcs.lublin.pl (A.D.-M.); malgorzata.seczkowska@umcs.pl (M.W.)

* Correspondence: agnieszka.chrzanowska@poczta.umcs.lublin.pl

Received: 10 July 2020; Accepted: 29 July 2020; Published: date

Structure Characterization of MCF Supports

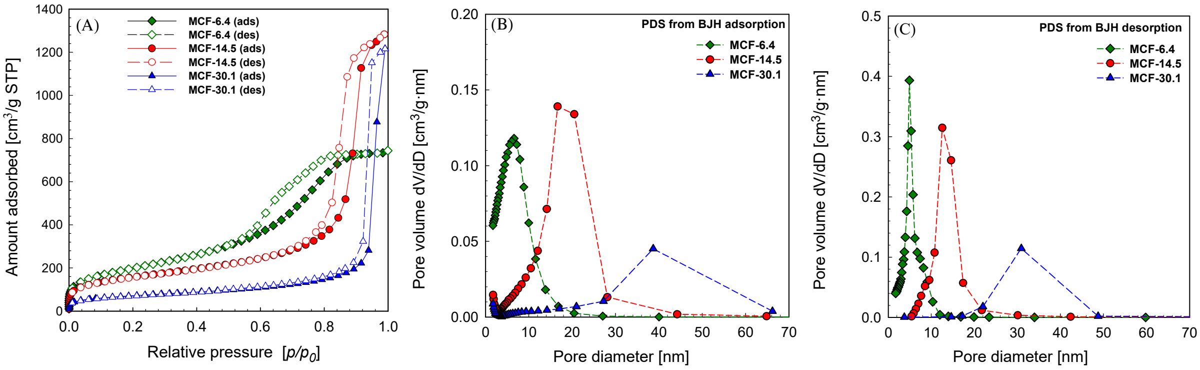

Figure S1. (**A**) Comparison of nitrogen adsorption/desorption isotherms for selected MCF materials with different pore sizes (MCF-6.4 nm, MCF-14.5 nm, MCF-30.1 nm). (**B**, **C**) Pore size distributions calculated by using BJH method for the adsorption and desorption branches of isotherms.

Adsorption Kinetics

Table S1. Relative standard deviations SD(c)/c_o for m-exp, FOE, SOE, MOE, f-FOE, f-SOE, F-MOE, McKay pore diffusion (PDM) and IDM model (Crank).

System	m-exp	FOE	SOE	MOE	f-FOE	f-SOE	f-MOE	IDM	PDM
LYS/MCF-6.4	0.337%	0.430%	0.389%	0.392%	0.246%	0.247%	2.242%	2.897%	4.495%
LYS/MCF-14.5	0.414%	4.587%	4.422%	3.499%	3.360%	2.625%	2.414%	7.196%	20.949%
LYS/MCF-30.1	0.723%	3.066%	3.033%	1.274%	2.478%	2.466%	3.776%	4.357%	22.187%
average	0.491%	2.694%	2.615%	1.722%	2.028%	1.779%	2.811%	4.817%	15.877%

The kinetic equations and models: multi-exponential equation (m-exp), first-order equation (FOE), second-order equation (SOE), mixed-order equation (MOE), fractal first-order equation (f-FOE), fractal second-order equation (f-SOE), fractal mixed-order equation (f-MOE), intraparticle diffusion model (IDM), McKay pore diffusion model (PDM).

Physicochemical Properties of Protein/MCF Composites

Structural Analysis

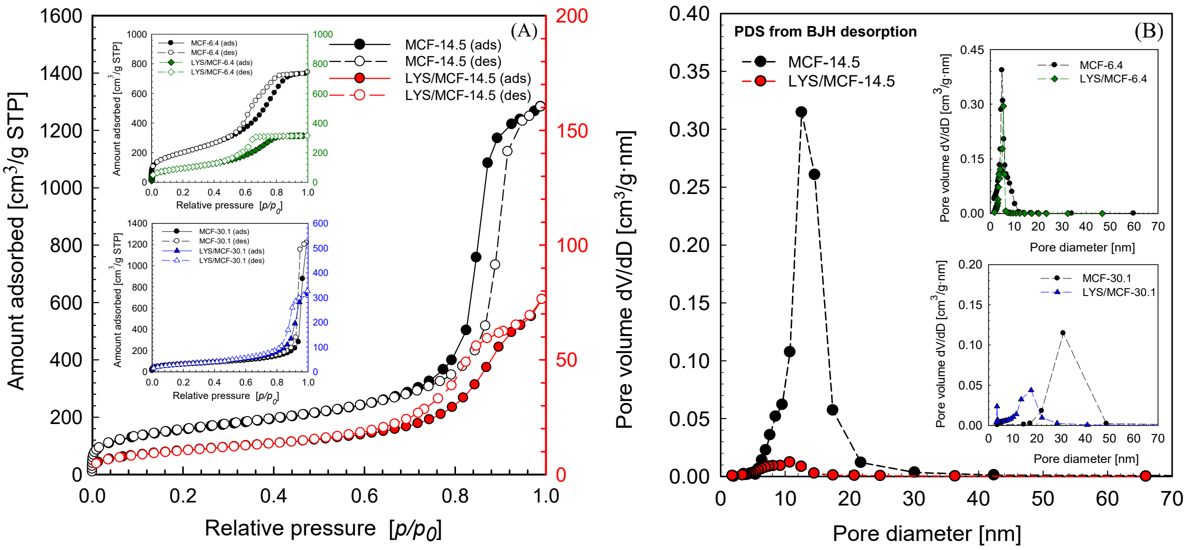

Figure S2. (**A**) Comparison of N_2 adsorption/desorption isotherms before and after LYS adsorption for MCF-14.5, and MCF-6.4, MCF-30.1 (inset plots). (**B**) Differential pore size distributions (PSDs) evaluated from the BJH model based on desorption data for pure MCF-14.5 support and covered by the LYS molecules. Inset is the pore size distributions for pure MCF-6.4, MCF-30.1 supports, and after LYS adsorption.

Acid-Base Properties

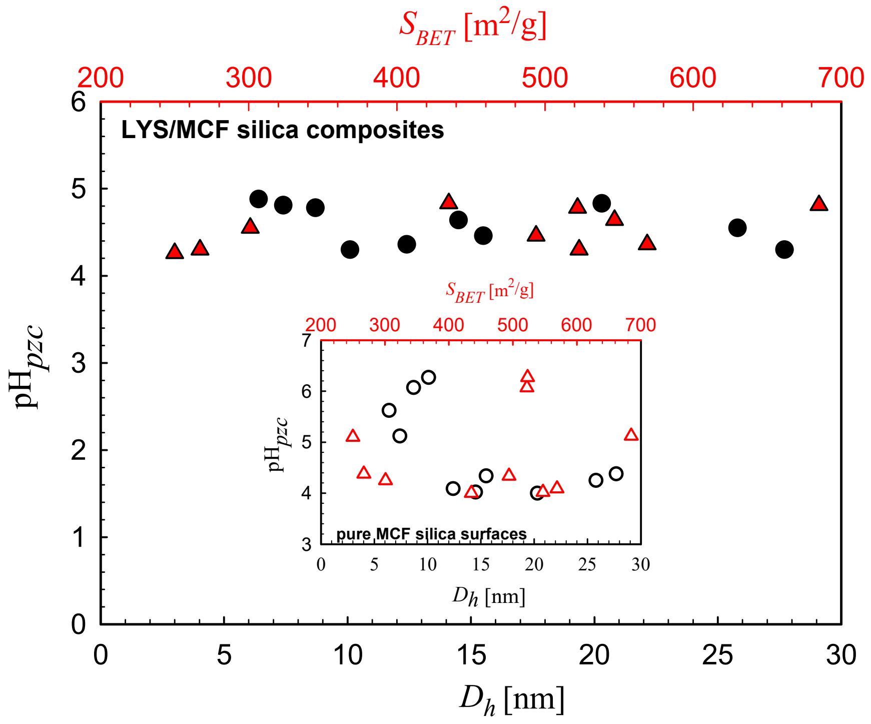

Figure S3. Influence of hydraulic pore diameter (D_h) and surface area (S_{BET}) on the point of zero charge (pH$_{pzc}$) of MCF materials after LYS adsorption. Inset: variations pH$_{pzc}$ as a function of D_h and S_{BET} for pure supports.

Synthesis of Mesocellular Foam

Table S2. Preparation conditions in MCF synthesis.

Material	Polymer Type	Polymer/TMB Mass Ratios [g/g]	Polymer/TEOS Mass Ratios [g/g]	Aging Temp./Time [°C/h]
MCF-6.4	PE9400	1:1	1:0.9	120/24
MCF-7.4	PE9400	1:1	1:0.9	122/24
MCF-8.7	PE9400	1:1	1:0.7	122/24
MCF-10.1	PE9400	1:1	1:1.8	120/24
MCF-12.4	PE9400	1:1	1:2.2	120/24
MCF-14.5	PE9400	1:1	1:2.2	110/144
MCF-15.5	P123	1:1	1:2.2	110/72
[a]MCF-20.3	P123	1:2.5	1:3	120/24
[a]MCF-25.8	P123	1:2.5	1:3	120/96
[a]MCF-27.7	P123	1:3.5	1:3	120/96
[a]MCF-30.1	P123	1:5	1:3	120/96

[a] The mineral agent NH_4F.
